# *Yeokwisan*, a Standardized Herbal Formula, Enhances Gastric Emptying via Modulation of the Ghrelin Pathway in a Loperamide-induced Functional Dyspepsia Mouse Model

**DOI:** 10.3389/fphar.2021.753153

**Published:** 2021-09-22

**Authors:** Seung-Ju Hwang, Jing-Hua Wang, Jin-Seok Lee, Hwa-Dong Lee, Tae-Joon Choi, Seo-Hyung Choi, Chang-Gue Son

**Affiliations:** ^1^ Liver and Immunology Research Center, Daejeon Oriental Hospital of Daejeon University, Daejeon, South Korea; ^2^ National Institute for Korean Medicine, Daejeon, South Korea; ^3^ Wooje IM Inc., Daejeon, South Korea; ^4^ Department of Internal Medicine, Gangnam Weedahm Korean Medical Hospital, Daejeon, South Korea

**Keywords:** functional dyspepsia, ghrelin, gastric emptying, herbal formula, interstitial cells of cajal

## Abstract

**Background:** Yeokwisan, a standardized herbal formula, has exhibited clinical benefit for patients suffering from refractory functional dyspepsia (FD) in Korea since 2016. However, data about the mechanism of action of this formula are yet not available.

**Aim of the study:** To evaluate and explore the effects of Yeokwisan on gastric emptying, a major symptom of functional dyspepsia, and its underlying mechanisms of action using a mouse model.

**Materials and methods:** BALB/C mice were pretreated with Yeokwisan (100, 200, and 400 mg/kg, po) or mosapride (3 mg/kg, po) for 5 days and then treated with loperamide (10 mg/kg, ip) after 20 h of fasting. A solution of 0.05% phenol red (500 μL) or diet of 5% charcoal (200 μL) was orally administered, followed by assessment of gastric emptying or intestinal transit. Plasma acyl-ghrelin (ELISA), C-kit (immunofluorescence and western blotting), nNOS (western blotting) and gastric contraction- and ghrelin-related gene/protein expression levels were examined in stomach and small intestine tissues.

**Results:** Loperamide injection substantially delayed gastric emptying, while Yeokwisan pretreatment (especially 200 and 400 mg/kg Yeokwisan) significantly attenuated this peristaltic dysfunction, as evidenced by the quantity of phenol red retained in the stomach (*p* < 0.05 or 0.01) and stomach weight (*p* < 0.05 or 0.01). The levels of plasma acyl-ghrelin and expression of gastric ghrelin-related genes, such as growth hormone secretagogue receptor (GHSR), ghrelin-O-acyltransferase (GOAT), adrenergic receptor β1 (ADRB1) and somatostatin receptor (SSTR), were significantly normalized (*p* < 0.05 or 0.01) by Yeokwisan (400 mg/kg). Yeokwisan (400 mg/kg) significantly tempered the loperamide-induced alterations in the c-kit and nNOS levels (*p* < 0.01) as well as the expression of contraction- and ghrelin-related genes, such as 5-HT4 receptor (5-HT4R), anoctamin-1 (ANO1), ryanodine receptor 3 (RYR3) and smooth muscle myosin light chain kinase (smMLCK), in the stomach, but not in the small intestine.

**Conclusion:** The present results showed the clinical relevance of Yeokwisan, in treating FD, especially in promoting gastric emptying but not small intestinal transit. The main mechanisms corresponding to these effects may involve the modulation of the ghrelin pathway and activation of interstitial cells of Cajal in stomach tissue.

## Introduction

Approximately one-fifth of the general population complains of dyspeptic symptoms, such bloating, anorexia, early satiety, and epigastric discomfort (Ford et al., 2015). Eighty percent of them cannot be explained either structurally or organically, and these symptoms are referred to as functional dyspepsia (FD) (Ford et al., 2020). FD accounts for 11–29.2% of the global prevalence of these symptoms, with differences among countries (Mahadeva and Goh, 2006). The economic burden of FD is estimated to be over 18 billion dollars per year in the United States (Lacy et al., 2013).

FD is diagnosed by symptom-based criteria, and the Rome IV criteria were most recently revised in 2016 (Stanghellini et al., 2016). FD is generally divided into three subtypes depending on the main symptoms: postprandial fullness and early satiety (postprandial distress syndrome, PDS), epigastric pain/burning symptoms (epigastric pain syndrome, EPS), and a combination of these symptoms (Asano et al., 2016). In Asia, the PDS subtype is known to be more prevalent than the EPS or combination type, especially in Korea and Japan (Lee and Chua, 2012).

The main causes of FD generally include abnormal gastrointestinal (GI) motility, visceral hypersensitivity, oversecretion of gastric acid, and *Helicobacter pylori* infection (Enck et al., 2017). Based on these etiological factors, prokinetic drugs, such as ghrelin receptor agonists, serotonin (5-hydroxytryptamine, 5-HT) receptor agonists, muscarinic receptor antagonists, proton pump inhibitors (PPIs), and *H. pylori* eradicating drugs are used to treat patients with FD (Ford et al., 2020). However, these treatments have clinical limitations, such as a high recurrence rate after cessation-, for example, the recurrence rate after cessation of acotiamide, a prokinetic, therapy, is a half (Shinozaki et al., 2020), and unexpected adverse effects, such as an increased risk of myocardial infraction and risk of ventricular arrhythmias after long-term use of PPIs or cisapride (a 5-HT receptor agonist) (De Maeyer et al., 2008; Shah et al., 2015).

On the other hand, herbal medicine has been used as a treatment option for patients with GI disorders. One study reported that approximately one-third of surveyed patients with functional GI disorders used herbal medicines (Lahner et al., 2013). Many patients with FD have chosen herbal products from traditional Korean medicine (TKM) and traditional Chinese medicine (TCM). Many studies have shown the therapeutic effects of multiherbal decoctions in clinical trials (Kim et al., 2014; Kim et al., 2021) and animal experiments (Jeon et al., 2019; Tan et al., 2020). In particular, the pharmacological theories of TKM and TCM emphasize the synergistic actions of multiherbal combinations for the treatment of multifactorial disorders, such as FD (Liu et al., 2013; Kim et al., 2021).

*Yeokwisan*, a standardized Korean herbal formula, is composed by six herbs including *Poncirus trifoliata* Rafinesque (*P. trifoliata*)*, Scutellaria baicalensis* Georgi (*S. baicalensis*)*, Glycyrrhiza uralensis* Fischer (*G. uralensis*), Massa medicata Fermentata*, Phyllostachys bambusoides* Sieb. et Zucc (*P. bambusoides*), and *Ostrea gigas* Thunberg (*O. gigas*). It has been prescribed for patients suffering from refractory FD including gastroesophageal reflux disease (GERD) in clinic since 2016. However, data about the mechanism of action this formula are yet not available. Accordingly, we aimed to evaluate and explore the effects and underlying mechanisms of this formula *in vivo* using a loperamide-induced FD mouse model.

## Materials and Methods

### Chemicals and Reagents

The following reagents and chemicals were obtained from Sigma-Aldrich (MO, United States): loperamide hydrochloride, mosapride citrate salt dihydrate, phenol red, sodium carboxymethyl cellulose (CMC-Na), sodium hydroxide, trichloroacetic acid (TCA), Tris base, sodium chloride, Triton X, 10% neutral formalin, calcium carbonate, calcium sulfate, catechin, chlorogenic acid, porcirin, naringin, rutin, benzaldehyde, aqueous mounting buffer, and 4′,6-diamidino-2-phenylindole dihydrochloride (DAPI).

Other reagents and chemicals were purchased from the following manufacturers: arabic gum (JUNSEI, Tokyo, Japan), activated charcoal power (YAKURI, Tokyo, Japan), Tween 20 (Glentham Life Science, Corsham, United Kingdom), skim milk (LPS solution, Daejeon, Korea), bovine serum albumin (GenDEPOT, TX, United States), hydrochloride (DUKSAN, Seoul, Korea), and hydroperoxide (SAMCHUN, Seoul, Korea).

### Preparation and Fingerprinting Analyses of *Yeokwisan*

*Poncirus trifoliata* Rafinesque (*P. trifoliata*)*, Scutellaria baicalensis* Georgi (*S. baicalensis*)*, Glycyrrhiza uralensis* Fischer (*G. uralensis*), Massa medicata Fermentata*, Phyllostachys bambusoides* Sieb. et Zucc (*P. bambusoides*), and *Ostrea gigas* Thunberg (*O. gigas*) were obtained from Weedahm Korean Hospital (Seoul, Korea), and all the herbs were approved by the Ministry of Food and Drug Safety (MFDS) in Korea. *O. gigas* Thunberg and others were extracted with boiling water and 60% EtOH solution, respectively. Then, these herbal medicine extracts were prepared by mixing them in certain proportions. The mixed formula was promptly stored at −70°C until use. Check Table 1 shows the yield and formula ratio of each herbal medicine.

**TABLE 1:** The yield and mixing portion of herbal medicines comprising *Yeokwisan*.

Herb	Extraction solvent	Yield (%)	Mixture portion (%)
*Poncirus trifoliata* Rafinesque	60% EtOH	17.2	16.4
*Scutellaria baicalensis* Georgi	60% EtOH	50.4	47.8
*Glycyrrhiza uralensis* Fischer	60% EtOH	30.0	21.4
Massa medicata Fermentata	60% EtOH	11.3	11.1
*Phyllostachys bambusoides* Sieb. et Zucc	60% EtOH	2.2	1.9
*Ostrea gigas* Thunberg	Boiled water	0.4	1.4

Fingerprinting analyses of *Yeokwisan* were conducted using high-performance liquid chromatography (HPLC). A total of 50 μg of *Yeokwisan* and 1 μg of each reference compound (naringin, baicalin, poncirin, baicalein, glycyrrhizic acid, and wogonin) were dissolved in 1 ml of 50% methanol, and the solution was filtered (0.2 μm). A 10 μL volume of each sample solution was injected into an Agilent 1,260 system, and separation was performed using a YMC-Triart C18 (5 μm, 4.6 × 250 mm, Agilent Technologies, CA, United States). The column was eluted at a flow rate of 1 ml/min and a wavelength of 230 nm using mobile phases A (0.05% phosphate in H_2_O) and B (acetonitrile including phosphate).

### Animals and Experimental Design

A total of one hundred-eight BALB/C male mice (6 weeks old; 19–21 g) were purchased from Daehanbio-link (Eumseong-gun, Chung-buk, Korea). These animals were maintained at room temperature (22 ± 2°C) and 60 ± 5% relative humidity under a 12-h light:12-h dark cycle. The mice were given free access to a commercial pellet diet (Daehanbio-link) and tap water.

After 7 days of acclimatization, the mice were randomly divided into three experimental sets: the first set was used to measure gastric emptying (n = 36), the second set was used to test intestinal motility (n = 36), and the third set was used to obtain tissues (stomach and small intestine) and blood samples (n = 36). Each set was divided into six groups (n = 6/group): the normal, control, three doses of *Yeokwisan* (100, 200, and 400 mg/kg), and mosapride groups. Commonly, the dose of *Yeokwisan* is 3 g per day in clinic for human adult, which is equivalent to 615 mg/kg of mouse according to animal equivalent dose calculation based on body surface (Food and Administration, 2005; Nair and Jacob, 2016). However, we have found that even though 400 mg/kg of *Yeokwisan* showed the significant prokinetic effect on stomach in our pilot experiment. Therefore, 400 mg/kg was set to the high dose in the present experiment.

*Yeokwisan*, mosapride and distilled water were orally treated separately in each corresponding group once a day for continuous 5 days. On the final day of the experiment, the mice were fasted for 20 h. Then, the mice were examined in accordance with the protocol for each experimental set, as follows: 1) gastric emptying test, 2) intestinal transit tests, and 3) biomolecular analysis. The corpus region in stomach and duodenum in small intestine (regions up to 5 cm from the pyloric sphincter) were used for biomolecule analysis.

The protocol was approved by the Institutional Animal Care and Use Committee of Daejeon University (Daejeon, Republic of Korea; Approval No. DJUARB 2021-013) and was conducted in accordance with the Guide for the Care and Use of Laboratory Animals, published by the National Institutes of Health (NIH, MD).

### Determination of Gastric Emptying Using Phenol Red and Stomach Weight and Area

The mice were fasted for 20 h and given free access to tap water. Except for those in the normal group, the mice were intraperitoneally injected with loperamide hydrochloride (10 mg/kg, dissolved in normal saline). After 30 min, all the mice were orally administered phenol red solution (500 μL/mouse). Phenol red was dissolved in 1.5% sodium carboxymethyl cellulose sodium (dissolved in distilled water) at a concentration of 0.05%. Thirty minutes after phenol red treatment, the mice were euthanized in a CO_2_ chamber (Jeungdo Bio and Plant, Seoul, Korea), and then, the stomachs were immediately removed and weighed. To measure the area of the stomach, all the stomachs were photographed, and then, the area of the stomach was calculated by ImageJ (NIH). In the experiments to determine gastric emptying, the choice of the phenol red solution volume and time point at which approximately 60% delayed gastric emptying was observed were established by our pilot experiment data (Supplementary Figure S1) and other protocols (Asano et al., 2016; Lee et al., 2016).

To measure the absorbance of the phenol red retained in the stomach, stomach samples were homogenized in 5 ml of 0.1 N sodium hydroxide solutions and 0.5 ml of 20% trichloroacetic acid. The homogenates were centrifuged at 3,000 rpm for 20 min, and then, 1 ml of supernatant was added to 4 ml of 0.5 N sodium hydroxide. Finally, the absorbance of these pink-colored solutions was determined at 560 nm by using a spectrophotometer.

The gastric emptying rates were calculated according to the following formula: gastric emptying (%) = (1-X/Y) * 100. X: Absorbance of stomach-retained phenol red, Y: Absorbance of naïve phenol red mixed with sodium hydroxide.

### Determination of Intestinal Transit Rate Using Charcoal Diet

To evaluate the intestinal transit rate, the mice were intraperitoneally injected with loperamide hydrochloride (10 mg/kg), except for the mice in the normal group. After 30 min, all the mice were orally administered 5% charcoal dissolved in 10% arabic gum (200 μL/mouse), a black semisolid paste, as previously described (Nunes Marona and Bastos Lucchesi, 2004). The mice were sacrificed 30 min after the charcoal diet treatment, and the intestinal transit was determined by measuring the distance of charcoal transit from the pylorus to the cecum by using ImageJ (NIH). The time points examined in these experiments involving treatment with charcoal diet and loperamide were established by our pilot experiment data (Supplementary Figure S2) and other protocols (Mittelstadt et al., 2005).

### Determination of Acylated Ghrelin Levels in Plasma by ELISA

Blood was immediately collected in K_2_-ethylenediaminetetraacetic acid (EDTA) tubes. After shaking for 15 min, blood was centrifuged at 3,000 rpm for 15 min. Then, PMSF was added to isolated plasma to prevent the degradation of acyl-ghrelin. To evaluate systemic acyl-ghrelin levels, plasma was measured by using a commercial acylated ghrelin ELISA kit (A05117, Bertin Pharma, France) according to the manufacturer’s protocol.

### Stomach and Small Intestine Protein Expression Analysis by Western Blotting

To determine the expression of C-kit and neuronal nitric oxide synthase (nNOS) in the stomach and small intestine, the stomach and small intestine were prepared in RIPA lysis buffer. The proteins were separated by 7.5% polyacrylamide gel electrophoresis and transferred to polyvinylidene fluoride (PVDF) membranes. After blocking in 5% skim milk for 1 h at room temperature, the membranes were incubated with primary antibodies, such as C-kit (0.1 μg/ml, AF1356, R&D Systems), nNOS (1:1,000, ab76067, Abcam) or α-tubulin (1:1,000, ab7291, Abcam) antibodies, overnight at 4°C. After washing with 0.1% TBS-T, the membranes were incubated with HRP-conjugated anti-goat (against C-kit, 1:2,500), anti-rabbit (against nNOS, 1:5,000), or anti-mouse (against α-tubulin, 1: 5,000) antibodies. These proteins were visualized using an enhanced chemiluminescence (ECL) advanced kit (Thermo Fisher Scientific, United States) and imaged using a FUSION Solo System (Vilber Lourmat, France). Protein expression was semiquantified using ImageJ (NIH).

### Stomach and Small Intestine Gene Expression Analysis by Quantitative Real-Time PCR

#### Ghrelin-Related Genes

Expression of ghrelin-related genes was analyzed by quantitative real-time PCR. The ghrelin-related genes were as follows: ghrelin, ghrelin-O-acyltransferase (GOAT), growth hormone secretagogue receptor (GHSR), adrenergic receptor β1 (ADRB1) and somatostatin receptor (SSTR).

#### Smooth Muscle Contraction-Related Genes

Regarding smooth muscle cell contraction, quantitative real-time PCR analysis was conducted to evaluate the expression of the following four smooth muscle cell contraction-related gene: 5-HT_4_ receptor (5-HT_4_R), anoctamin-1 (ANO1), ryanodine receptor 3 (RYR3) and smooth muscle cell myosin light chain kinase (smMLCK).

#### Quantitative Real-Time PCR Performance

Total RNA was extracted from the stomach and small intestine tissues using QIAzol reagent (QIAGEN, Germany). cDNA was synthesized from total RNA (2 ug) using a High-Capacity cDNA Reverse Transcription Kit (4368814, Thermo Fisher Scientific, United States). Quantitative real-time PCR was performed using SYBR Green PCR Master Mix (Applied Biosystems, United States) and primers as described in Table 2. Gene expression data were analyzed using the IQ5 PCR Thermal Cycler (Bio-Rad, United States).

**TABLE 2:** Summary for gene sequence.

Gene	Sense (5´→3′)	Anti-sense (5´→3′)
5-HT_4_R	ATG GTC AAC AAG CCC TAT GC	AGG AAG GCA CGT CTG AAA GA
ANO1	GGT GTC GGG TTT GTG AAG AT	TGC ACG TTG TTC TCT TCA GG
RYR3	GGC CAA GAA CAT CAG AGT GAC TAA	TCA CTT CTG CCC TGT CAG TTT C
smMLCK	AGA AGT CAA GGA GGT AAA GAA TGA TGT	CGG GTC GCT TTT CAT TGC
Ghrelin	TCC AAG AAG CCA CCA GCT AA	AAC ATC GAA GGG AGC ATT GA
GHSR	CTA TCC AGC ATG GCC TTC TC	AAG ACG CTC GAC AC CCA TAC
GOAT	ATT TGT GAA GGG AAG GTG GAG-	CAG GAG AGC AGG GAA AAA GAG
ADRB1	GAA GGC GCT CAA GAC ACT GG	CCA GGT CGC GGT GGA A
SSTR	GGC GAA ATG CGT CCC AG	CGG AGT A GA TGA AAG AGA TCA GGA
GAPDH	CAT GGC CTT CCG TGT TCC T	CCT GCT TCA CCA CCT TCT TGA

5-HT_4_ receptor, 5-HT_4_R; anoctamin-1, ANO1; ryanodine receptor 3, RYR3; smooth muscle cell myosin light chain kinase, smMLCK; growth hormone secretagogue receptor, GHSR; ghrelin-O-acyltransferase, GOAT; adrenergic receptor β1, ADRB1; somatostatin receptor, SSTR.

### Stomach and Small Intestine Immunofluorescence Staining Analysis

Paraffin sections of stomach and small intestine tissues (4 μm) were dried at 60°C for 15 min. The sections were then subjected to deparaffinization with xylene and to rehydration with ethanol (with 100, 95, 85, 70, and 50% ethanol and tap water for 3 min each). Antigens were retrieved by incubating with 10 mM sodium citrate buffer for 10 min. After washing three times, nonspecific binding affinity was blocked for 1 h using normal goat serum, and then, the slides were incubated with the anti-c-kit antibody (1:200) overnight at 4°C. After washing, the slides were incubated with a goat anti-rabbit Alexa Fluor-488 conjugated secondary antibody (1:200) for 1 h at RT. After washing three times for 10 min, the slides then were incubated with DAPI (1 μg/ml) for 2 min at RT in the dark. The c-kit signal was observed using an Axio-phot microscope (Carl Zeiss, Germany). The c-kit protein expression was semiquantified using ImageJ (NIH).

### Statistical Analysis

The data are expressed as the mean ± standard deviation (SD) or fold changes in means. Statistical significance was determined by using one-way analysis of variance (ANOVA) followed by Dunnett’s test. In all analyses, *p* < 0.05 was considered to indicate statistical significance.

## Results

### Fingerprinting Analysis of *Yeokwisan*

**FIGURE 1:**
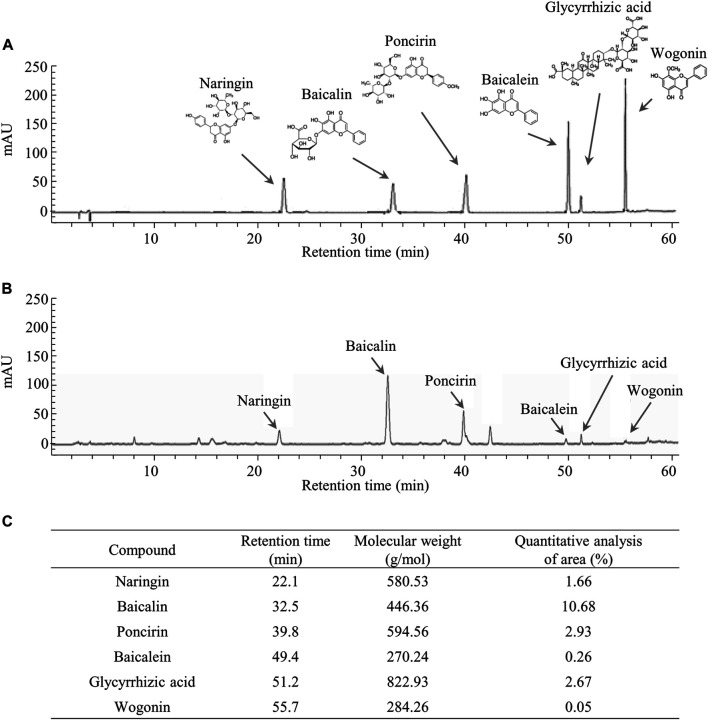
Fingerprinting analysis of *Yeokwisan*. Chemical constitutions and quantitative analysis of *Yeokwisan* using high-performance liquid chromatography (HPLC). Six reference standards **(A)** and *Yeokwisan* **(B)** were subjected to UHPLC analysis. Quantitative analysis of *Yeokwisan* was conducted **(C)**.

The six compounds, namely naringin, baicalin, poncirin, baicalein, glycyrrhizic acid, and wogonin were detected at retention times of 22.1, 32.5, 39.8, 49.4, 51.2, and 55.7 min, respectively, in the tested samples. Semiquantitative analysis using the standard curves of the reference compounds showed *Yeokwisan* contained 1.66% naringin, 10.68% baicalin, 2.93% poncirin, 0.26% baicalein, 2.67% glycyrrhizic acid, and 0.05% wogonin (Figure 1).

### *Yeokwisan* Reversed the Delayed Gastric Emptying Caused by Loperamide

**FIGURE 2:**
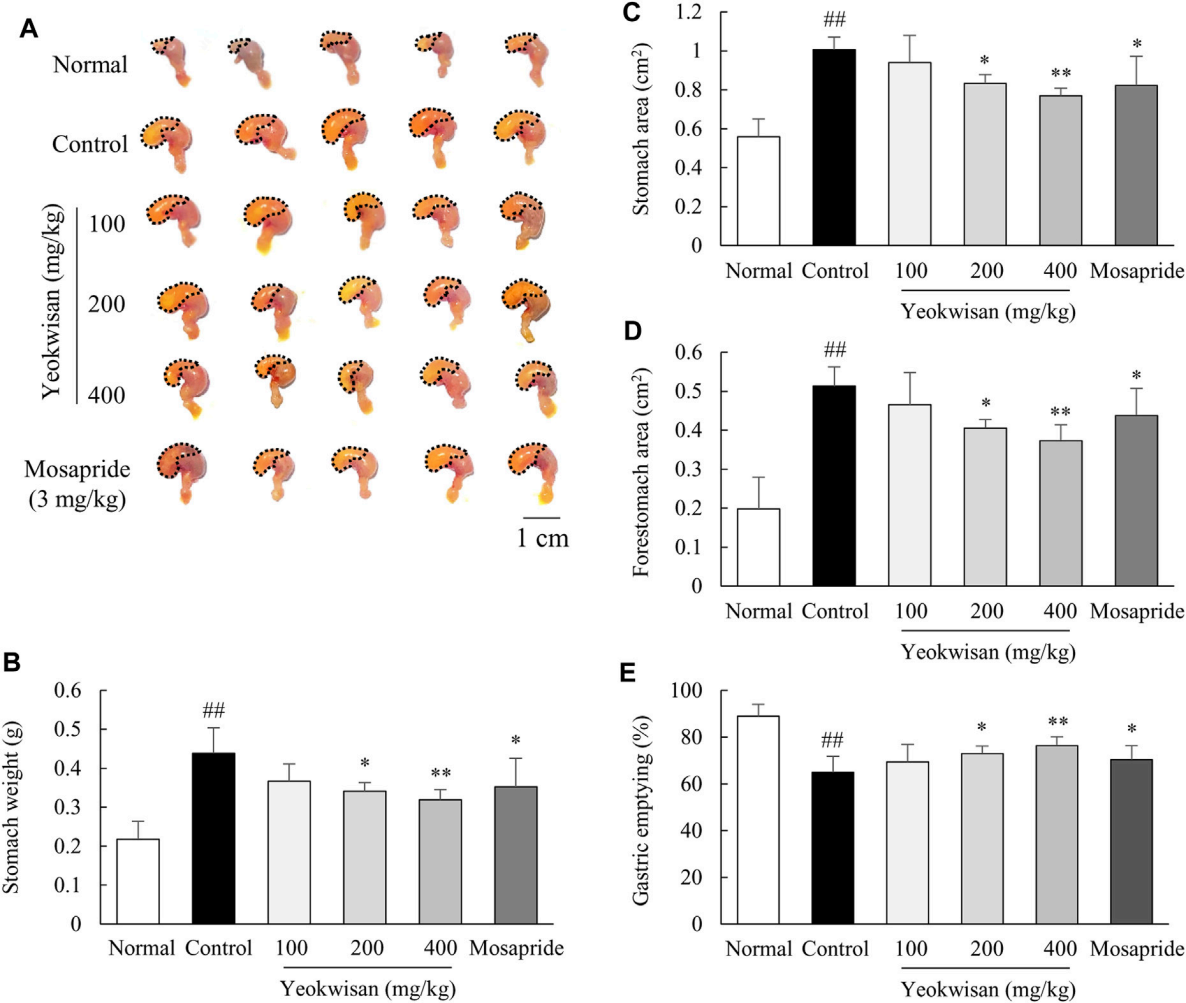
Effects of *Yeokwisan* on gastric emptying. For 5 days, the mice (n = 6/group) were orally administered *Yeokwisan* (100, 200 and 400 mg/kg) or mosapride (3 mg/kg) and then intraperitoneally injected with loperamide (10 mg/kg). After the administration of phenol red, visual observation **(A)**, stomach weight **(B)**, stomach area **(C)**, forestomach area **(D)** and gastric emptying **(E)** were assessed. The data are presented as the mean ± SEM. ##*p* < 0.01 compared with the normal group; *p < 0.05, **p < 0.01 compared with the control group.

As expected, loperamide injection inhibited the passage of phenol red, leading to fullness of the stomach, and this effect was significantly attenuated by pretreatment with *Yeokwisan* (*p* < 0.05 for 200 mg/kg and *p* < 0.01 for 400 mg/kg), as observed by the naked eye (Figure 2A), measurement of stomach weight (Figure 2B), calculation of whole and forestomach area (Figures 2C,D), and quantification of the amount of phenol red retained in the stomach (Figure 2E). The effect of mosapride was comparable to that of 200 mg/kg *Yeokwisan*.

### *Yeokwisan* did Not Affect Intestinal Transit

**FIGURE 3:**
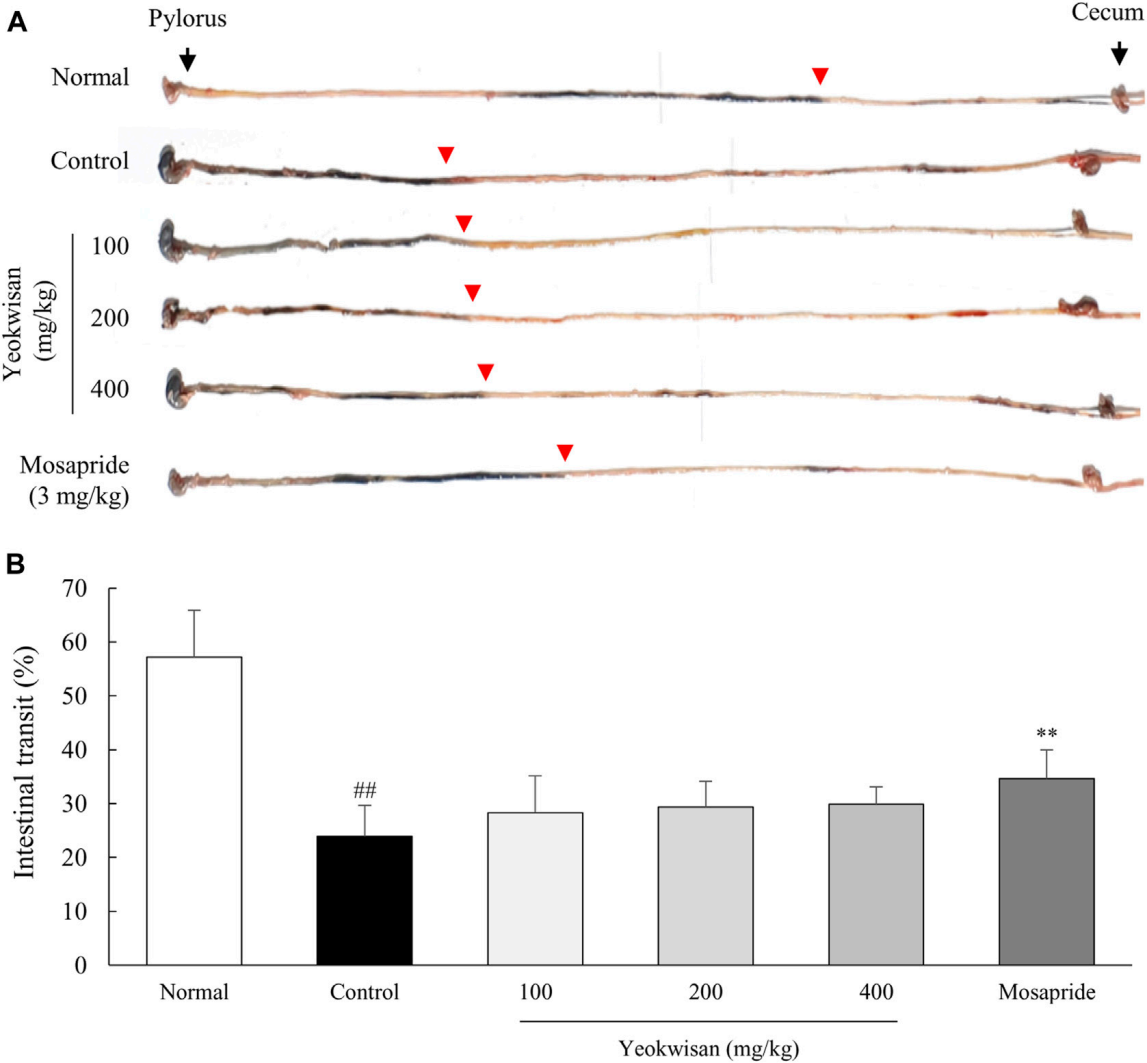
Effects of *Yeokwisan* on small intestinal transit. For 5 days, the mice (n = 6/group) were orally administered *Yeokwisan* (100, 200 and 400 mg/kg) or mosapride (3 mg/kg) and then intraperitoneally injected with loperamide (10 mg/kg). Thirty minutes after the administration of charcoal diets, the transit distance of the diet was measured **(A)** and quantified **(B)**. Red arrowheads indicate how much charcoal diet moved into the cecum. The data are presented as the mean ± SEM. ##*p* < 0.01 compared with the normal group; **p < 0.01 compared with the control group.

Loperamide treatment also notably decreased intestinal transit, and mosapride significantly attenuated this effect. Unexpectedly, *Yeokwisan* did not achieve a significant improvement of intestinal transit (*p* > 0.05 for all doses), even though it slightly accelerated the passage of the charcoal diet (Figures 3A,B).

### *Yeokwisan* Upregulated the Expression of C-Kit in the Stomach

**FIGURE 4:**
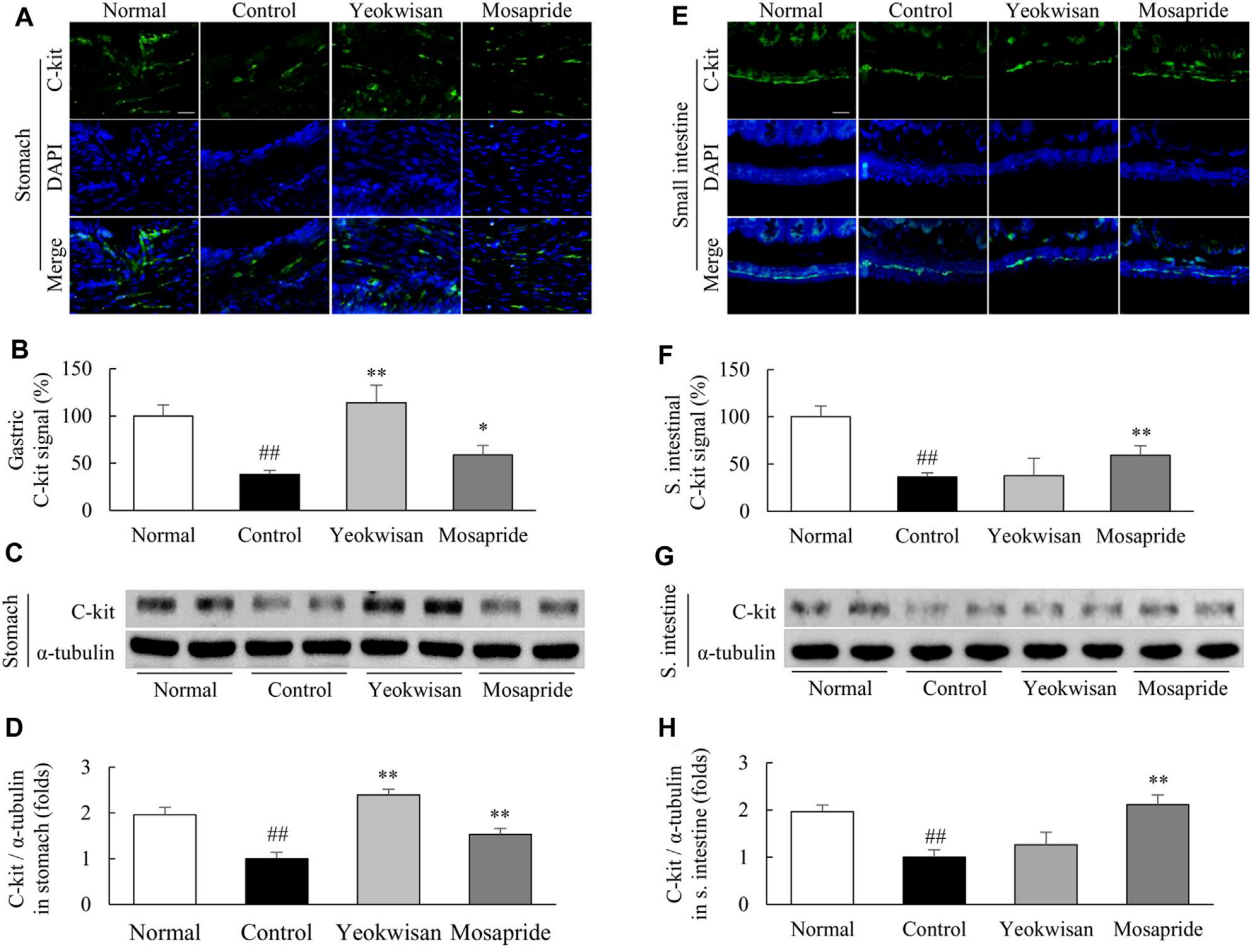
Effects of *Yeokwisan* on C-kit protein expression in the stomach and small intestine tissues. C-kit protein expression was semiquantitatively measured using immunofluorescence in the stomach **(A,B)** and small intestine **(E,F)** and semiquantitatively measured using western blotting in the stomach **(C,D)** and small intestine **(G,H)**. Immunofluorescence was observed under an optical microscope (200× magnification), and the scale bar indicates 50 μm. The *Yeokwisan* group was treated with a dose of 400 mg/kg in these results. The data are presented as the mean ± SEM. ##*p* < 0.01 compared with the normal group; *p < 0.05, **p < 0.01 compared with the control group.

Loperamide radically suppressed C-kit expression in the stomach and small intestine, while *Yeokwisan* pretreatment significantly attenuated these alterations in the stomach tissue, as evidenced by immunohistochemistry (*p* < 0.01, Figures 4A,B) and protein assays (*p* < 0.01, Figures 4C,D). Interestingly, these effects of *Yeokwisan* were not observed in small intestinal tissues. Mosapride, however, showed positive effects both the stomach and small intestine (*p* < 0.01, Figures 4E–H).

### *Yeokwisan* Upregulated the Expression of Proteins and Genes Associated With GI Motility

**FIGURE 5:**
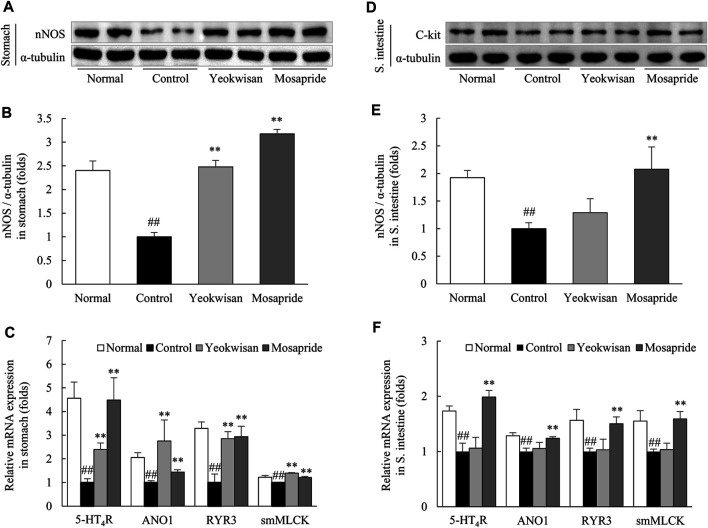
Effects of *Yeokwisan* on the expression of the nNOS protein and contraction-related genes in stomach and small intestine tissues. Western blotting analysis of nNOS and semiquantification of the data were performed in stomach **(A,B)** and small intestine **(D,E)** tissues. mRNA expression analyses for four GI motility-associated genes were conducted on stomach **(C)** and small intestine **(F)** tissues. The *Yeokwisan* group was treated with a dose of 400 mg/kg in these results. The data are presented as the mean ± SEM. ##*p* < 0.01 compared with the normal group; **p < 0.01 compared with the control group. nNOS, neuronal nitric oxide synthase; 5-HT_4_R, 5-HT_4_ receptor; ANO1, anoctamin-1; RYR3, ryanodine receptor 3; smMLCK, smooth muscle myosin light chain kinase.

In the protein assay, *Yeokwisan* pretreatment significantly attenuated the notable loperamide-induced suppression of nNOS expression in the stomach (*p* < 0.01, Figures 5A,B), but not in the small intestine (Figures 5D,E). In addition, loperamide notably lowered the expression of smooth muscle contraction-related genes, such as 5-HT_4_R, ANO1, RYR3, and smMLCK, in the stomach and small intestine. These alterations were significantly attenuated by *Yeokwisan* in the stomach (*p* < 0.01, Figure 5C) but not in the small intestine (Figure 5F). Mosapride treatment upregulated the expression of these protein and genes in both the stomach and small intestine (*p* < 0.01).

### *Yeokwisan* Increased the Plasma Ghrelin Concentration and Affected Ghrelin-Related Gene Expression

**FIGURE 6:**
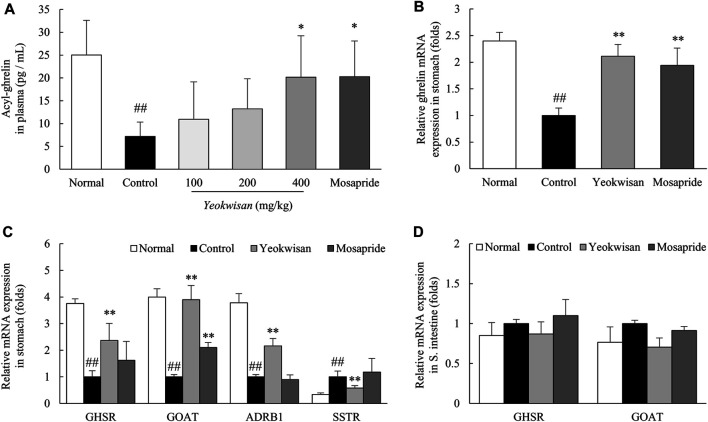
Effects of *Yeokwisan* on the level of ghrelin in plasma, and ghrelin-related genes in stomach and small intestine tissues. The level of acyl-ghrelin in plasma was determined using ELISA **(A)**. mRNA expression analyses of ghrelin in stomach tissue **(B)** and ghrelin-associated genes in the stomach **(C)** and small intestine **(D)** were performed. The *Yeokwisan* group was treated with a dose of 400 mg/kg in **(B–F)**. The data are presented as the mean ± SEM. ##*p* < 0.01 compared with the normal group; *p < 0.05, **p < 0.01 compared with the control group. GHSR, growth hormone secretagogue receptor; GOAT, ghrelin-O-acyltransferase; ADRB1, adrenergic receptor β1; SSTR, somatostatin receptor.

Loperamide treatment dramatically lowered the concentration of acylated ghrelin in plasma, whereas *Yeokwisan* (especially 400 mg/kg *Yeokwisan*) significantly ameliorated this change in concentration (*p* < 0.05, Figure 6A). This effect was supported by the gene expression of ghrelin in the stomach tissue (*p* < 0.01, Figure 6B). Loperamide also downregulated the gene expression of GHSR, GOAT and ADRB1 but upregulated the expression of SSTR in the stomach, while these alterations were significantly attenuated by *Yeokwisan* pretreatment (*p* < 0.05 or *p* < 0.01, Figure 6C). As expected, the expression of these genes (GHSR and GOAT) in the small intestine tissue was not altered by loperamide or *Yeokwisan* (Figure 6D). Mosapride showed similar effects to *Yeokwisan* for almost all parameters, except for GHSR, ADRB1, and SSTR gene expression in stomach tissue.

## Discussion

To investigate the pharmaceutical potential of *Yeokwisan* in the treatment of FD and its underlying mechanisms, we herein used a loperamide-induced FD mouse model. Loperamide, a μ2-opioid receptor agonist, suppresses the activity of the GI myenteric plexus, which decreases the tone of the circular and longitudinal smooth muscles of the GI tract (Katzung et al., 2004; Chen et al., 2012). Clinically, loperamide is used to treat diarrhea, and its adverse effects include abdominal pain, nausea, dyspepsia, and constipation (Hanauer, 2008). Thus, high doses of loperamide have been used to establish animal models of FD and constipation in preclinical studies (Jeon et al., 2019; Li et al., 2021).

As expected, our study showed that a single dose of loperamide injection (10 mg/kg, peritoneally) notably delayed both gastric emptying and intestinal transit (Figures 2, 3). Moreover, the administration of *Yeokwisan* (especially 200 and 400 mg/kg *Yeokwisan*) significantly attenuated this delayed gastric emptying, as evidenced by stomach weight, extended stomach volume and quantification of the amount of phenol red retained in the stomach (Figures 2A–E). These effects were very similar to those of mosapride, a positive control agent in the present study. 5-HT and its receptors are involved in the regulation of smooth muscle contraction, and 5-HT_4_R agonists such as mosapride are the main options for the treatment of functional disorders with impaired GI motility (Yang et al., 2017). In our results, both *Yeokwisan* and mosapride significantly restored the loperamide-induced downregulation of 5-HT_4_R gene expression in stomach tissue (Figure 5C). Abnormally suppressed gastric motility is one of the main causes of FD, and it has a negative impact, particularly on the PDS type of FD compared to the EPS type (Tack et al., 1998; Tack et al., 2002). Approximately 30% of all FD patients and 66% of all PDS patients showed delayed gastric emptying that was related to symptoms of postprandial fullness and early satiety and sometimes to gastroparesis-like symptoms, including vomiting and nausea (Talley et al., 2001; Sarnelli et al., 2003).

On the other hand, ghrelin, called a “hunger hormone”, has attracted attention as a key player in GI motility and as a therapeutic target for FD treatment (Yagi et al., 2013). Several studies found that ghrelin levels were significantly lower in patients with FD than in healthy volunteers (Takamori et al., 2007; Lee et al., 2009b). Both clinical studies (Murray et al., 2005; Binn et al., 2006) and animal studies (Trudel et al., 2002; Qiu et al., 2008) have shown that ghrelin binds to its receptor, GHSR, which consequently leads to the promotion of gastric peristalsis and passage of a meal. Ghrelin cells (G cells) of the stomach produce ghrelin in the form of des-acyl ghrelin, which is then converted into the active form (acyl-ghrelin) by ghrelin O-acyltransferase (GOAT) before being released into the blood (Yagi et al., 2013). As expected, loperamide injection drastically lowered the plasma level of acyl-ghrelin, while the reduction of acyl-ghrelin was significantly normalized by *Yeokwisan* (especially 400 mg/kg) (Figure 6A). This effect was also supported by the expression levels of genes related to the production of ghrelin (Figure 6B) and its acylation enzyme (GOAT) in stomach tissue (Figure 6C). The production and secretion of ghrelin is stimulated by various hormones, such adrenaline and somatostatin, which bind to receptors of the G cell membrane (Engelstoft et al., 2013). We confirmed that *Yeokwisan* activated a representative excitatory receptor (ADRB1) but suppressed an inhibitory receptor (SSTR) related to ghrelin production (Figure 6C).

It is well-known that GI motility is a continuous and repetitive process of segmental and peristaltic contractions and relaxations, in which interstitial cells of Cajal (ICCs) play a central role as pacemakers that generate electrical slow waves then transfer the these slow wave to the smooth muscle cells (Sanders et al., 2014). Some clinical studies have suggested that dysfunction or loss of ICCs causes abnormal GI motility-related diseases, including FD (Forster et al., 2005; Farrugia, 2008). To explore the involvement of ICCs in the gastric emptying effect of *Yeokwisan,* we evaluated the protein expression level of C-kit, a representative parameter of ICCs, in stomach tissue. As expected, administration of *Yeokwisan* significantly normalized the loperamide-induced loss of C-kit signal as shown by immunohistological findings and protein assays (Figures 4A–D). In fact, ghrelin binds to its receptor (GHSR) on ICCs and sequentially activates ICCs-derived electrical slow waves, as evidenced by *in vivo* and *in vitro* experiments (Yang et al., 2014; Kim and Kim, 2019). We also found that *Yeokwisan* upregulated the gene expression of GHSR in stomach tissue (Figure 6C). Slow wave generation and delivery by activated ICCs are regulated by several key molecules; ANO1 and RYR3 increase Ca^2+^ concentrations inside ICCs and generate intracellular Ca^2+^ waves, respectively (Sanders et al., 2006; Sanders et al., 2012). Then, the generated Ca^2+^ waves deliver electric signals to neighboring smooth muscle cells via gap junctions, which causes the contraction of smooth muscle by active smMLCK (Garcia et al., 1997). *Yeokwisan* administration attenuated the loperamide-induced changes in the expression of above-mentioned genes in stomach tissue (Figure 5C). In addition, regarding the relaxation process of smooth muscle in the GI tract, the nNOS-mediated production of nitric oxide (NO) is vitally important, as NO is retrograde neurotransmitter in synapses of vagus nerves (Terauchi et al., 2005). In clinical and animal studies, the administration of the NOS inhibitor, N^G^-nitro-L-arginine methyl ester, delayed gastric emptying, and nNOS^−/-^ mice also exhibited delayed gastric emptying (Plourde et al., 1994; Tack et al., 2002; Micci et al., 2005). In our study, *Yeokwisan* treatment significantly restored the loperamide-induced downregulation of nNOS protein expression in stomach tissue (Figures 5A,B). These results suggest that the pharmacological function of *Yeokwisan* is mainly associated with the modulation of ghrelin and ICCs activity in stomach tissue.

On the other hand, *Yeokwisan* did not improve intestinal transit as different from stomach (Figures 3A,B). These effects of *Yeokwisan* was very intriguing because prokinetics, such as mosapride, cisapride and acotiamide, generally work on the whole GI tract, both stomach and intestine (Tack et al., 2019). These results might indicate the not involvement of μ2-opioid receptor as the main mechanisms of *Yeokwisan,* because loperamide is an agonist of μ2-opioid receptor, suppressing the motility both in intestine and stomach (Chen et al., 2012). In the present study, we found *Yeokwisan* only promotes gastric motility via ghrelin-related pathway, but not intestinal transit (Figures 2, 3, 6). So, we propose the μ2-opioid receptor might not be markedly changed by *Yeokwisan*. In our study, mosapride accelerated the transit in the stomach (Figure 2) and small intestine (Figure 3). In our previous study, an herbal formula, *Banha-sasim-tang*, also promoted motility in both the stomach and small intestine (Jeon et al., 2019). Nevertheless, *Yeokwisan* changed potential GI motility-related proteins and genes as a different pattern between stomach and intestine. Briefly, *Yeokwisan* did not recover the GI motility-related proteins (C-kit and nNOS, Figures 4E–H, 5D,E) and genes (5-HT_4_R, ANO1, RYR3, and smMLCK, Figure 5F) expression, ghrelin-related genes (GHSR and GOAT, Figure 6D) expression in intestine, rather than stomach. As we known, G cells dominantly exist in stomach (Sakata and Sakai, 2010). Our data also showed that *Yeokwisan* significantly elevated ghrelin gene expression in stomach as compared to loperamide-treated group (Figure 6B). Therefore, it was proposed that ghrelin might be a key modulator on prokinetic effects in stomach by *Yeokwisan*. From the perspective of diarrhea-inducing adverse effects or clinical limitations of prokinetics (Quigley, 2015; Tack et al., 2019), these pharmacological characteristics of *Yeokwisan* may indicate the proper applicable spectrum of FD patients. Both FD and irritable bowel syndrome (IBS) are functional GI disorders (FGIDs) that are frequently comorbid. According to one study, 32% of patients with FD had overlapping IBS, and 37% of patients with IBS had FD symptoms (Suzuki and Hibi, 2011). IBS includes two major subtypes, the constipation-dominant or diarrhea-dominant type, and 16.6% of patients with diarrhea-dominant type (but 11% of patients with the constipation type) also had FD (Choi et al., 2017). In present study, Yeokwisan promote the gastric motility via modulation of GI-motility-related molecules (Figures 2, 4A–D, 5A–C), not in the small intestine (Figures 3, 4E–H, 5D–F). Because of these gastric-specific properties of *Yeokwisan,* it may be further beneficial for FD patients with overlapping diarrhea-type IBS.

Due to the clinical limitations of currently available drugs for FD including the relatively high recurrence rate after cessation of 51% (Shinozaki et al., 2020), herbal resources have received attention in drug development (Tan et al., 2020). We herein proved the anti-FD activity of *Yeokwisan,* a standardized formula consisting of a six-herbal mixture including *P. trifoliata*, *S. baicalensis, G. uralensis,* Massa medicata fermentata*, P. bambusoides, O. gigas* (Table 1). Previous animal studies reported beneficial effects of two composing herbs, *P. trifoliata,* and *S. baicalensis,* on dysfunction of GI motility in atropine-induced (Lee et al., 2005), and ritonavir-induced (Mehendale et al., 2007) conditions, respectively. In particular, *G. uralensis* significantly improved the symptoms of patients with FD in a clinical study (Raveendra et al., 2012). According to the Korean herbal pharmacopoeia (Food and Adiministration, 2012), *Yeokwisan* has been standardized using six compounds: baicalein and baicalin from *S. baicalensis*, naringin and poncirin from *P. trifoliata*, and glycyrrhizic acid and wogonin from *G. uralensis* (Figures 1A–C). Among these compounds, baicalin and baicalein showed antidepressive effects in stress-induced depression rodent models (Lee et al., 2013; Liu et al., 2019). The depressive mood or dysregulation of 5-HT is well-known to inhibit peristatic movement in the GI tract (Kheder et al., 2018), while patients with FD complain of high comorbidity of depression (Esterita et al., 2021). Gastroprotective effects of naringin, wogonin and poncirin were also reported in gastric ulcer, mucosal damage, and gastritis rat models (Park et al., 2004; Lee JH. et al., 2009; Emin and Volkan, 2019). In particular, naringin improved delayed GI transit by activating ghrelin receptor in a laparotomy-induced rat model (Jang et al., 2013). These previous data support our beneficial effects of *Yeokwisan*.

**FIGURE 7:**
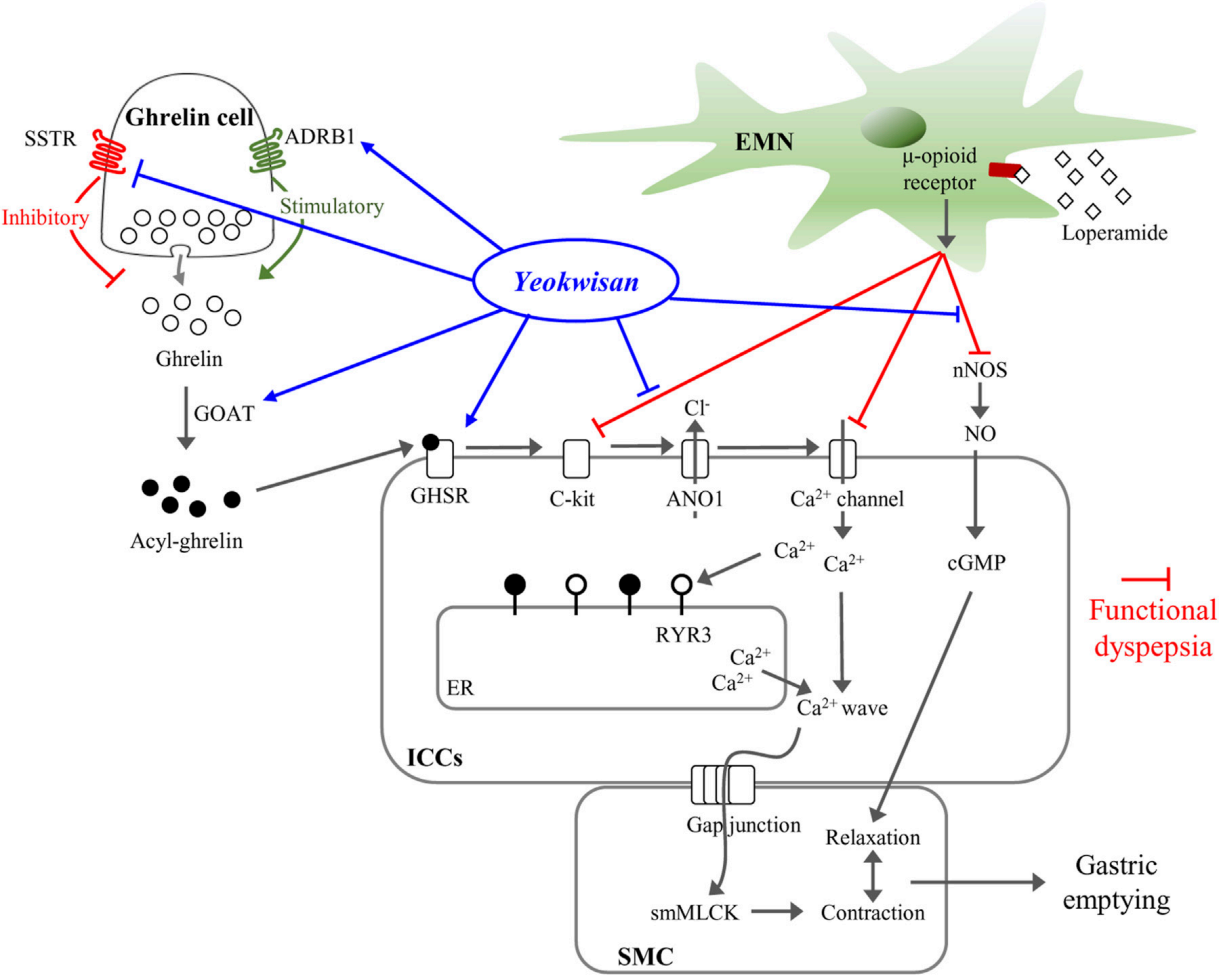
Summary for mechanism of *Yeokwisan* on stomach tissue in loperamide-induced functional dyspepsia model. ADRB1, adrenergic receptor β1; ANO1, anoctamin-1; cGMP, cyclic guanosine monophosphate; EMN, enteric motor neuron; ER, endoplasmic reticulum; GHSR, growth hormone secretagogue receptor; GOAT, ghrelin-O-acyltransferase; ICCs, interstitial cells of Cajal; nNOS, neuronal nitric oxide synthase; NO, nitric oxide; RYR3, ryanodine receptor 3; SSTR, somatostatin receptor; smMLCK, smooth muscle myosin light chain kinase; SMC, smooth muscle cell.

Accordingly, we expected the synergistic effects of *Yeokwisan*, a mixture of six herbs on gastric emptying, may supporting the clinical relevance of this drug for treatment of patients with dyspepsia and GERD (Figure 7). Although we cannot explain the underlying mechanisms, we propose that the stomach-specific effect on peristaltic movement resulted from the mixture of multiple herbs or compounds, which modulate the expression of ghrelin-related genes (Ghrelin, GHSR, GOAT, ADRB1, and SSTR) only in the stomach (Figures 6B,C) but not in the small intestine (Figure 6D). The lack of identification of active compounds is a limitation of the present study. Further research is needed to address these issues. In addition, we have to further investigate the ideal formulation of these multi-herbal combinations to maximize stomach-specific activity and its underlying mechanisms.

Taken together, our present data showed the clinical effect of *Yeokwisan* on FD in a loperamide-induced functional dyspepsia mouse model. The main mechanisms corresponding to these effects may involve the modulation of the ghrelin pathway, including activation of ICCs in stomach tissue. In particular, we found that *Yeokwisan* improves gastric emptying via stomach-specific effects on GI motility.

## Data Availability

The original contributions presented in the study are included in the article/Supplementary Material, further inquiries can be directed to the corresponding authors.
