# Supplementary material for: Yeokwisan, a Standardized Herbal Formula, Enhances Gastric Emptying via Modulation of the Ghrelin Pathway in a Loperamide-induced Functional Dyspepsia Mouse Model
Source: Front Pharmacol. 2021 Sep 22;12:753153. doi: 10.3389/fphar.2021.753153 (PMC8493126; doi:10.3389/fphar.2021.753153)
Supplement: Supplementary file 1 [file Presentation1.PPTX]

## Slide 1
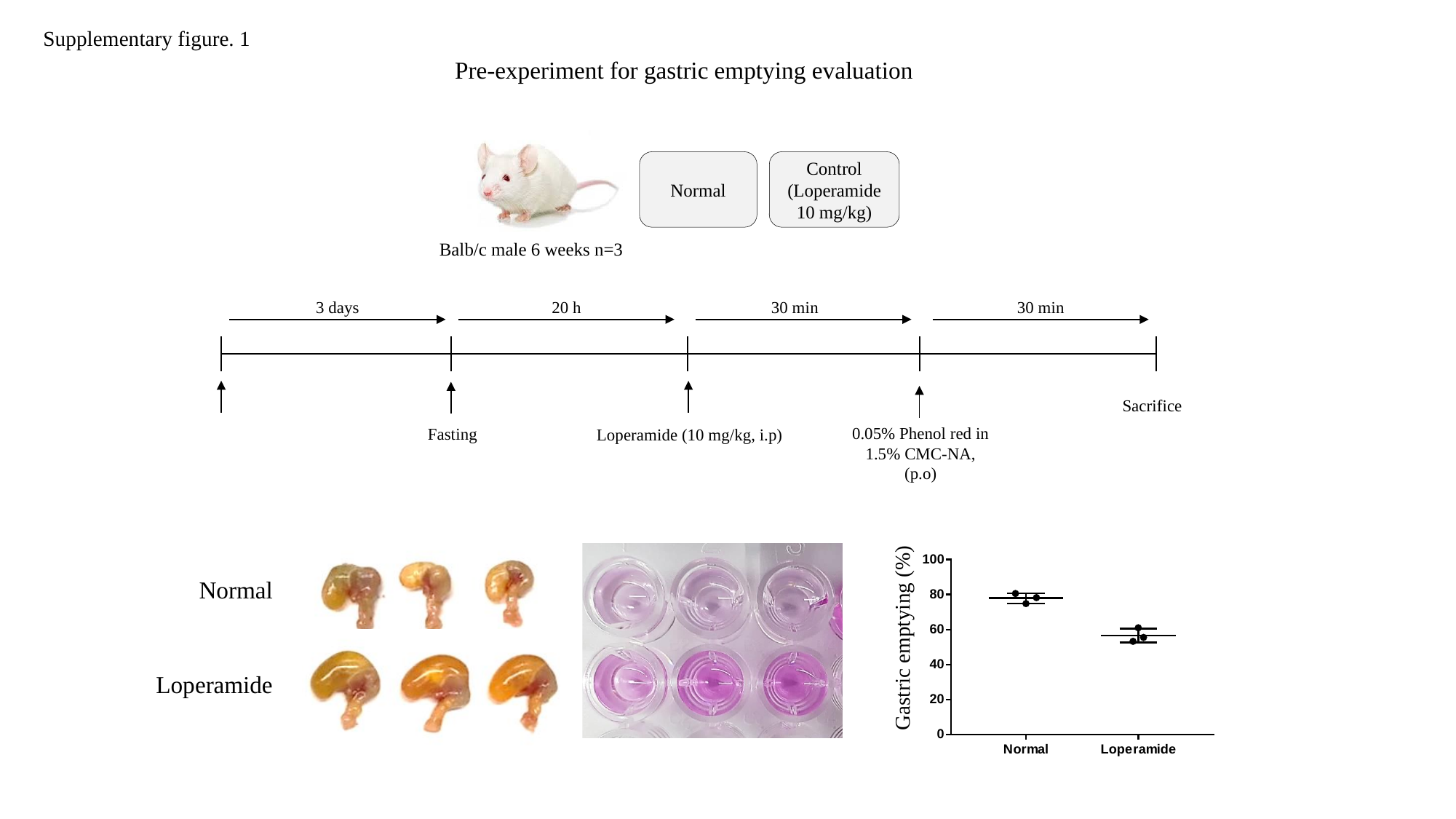

Supplementary figure. 1
Pre-experiment for gastric emptying evaluation
Normal
Control
(Loperamide 10 mg/kg)
Balb/c male 6 weeks n=3
30 min
3 days
20 h
30 min
Sacrifice
Fasting
 Loperamide (10 mg/kg, i.p)
0.05% Phenol red in 1.5% CMC-NA, (p.o)
Normal
Gastric emptying (%)
Loperamide

## Slide 2
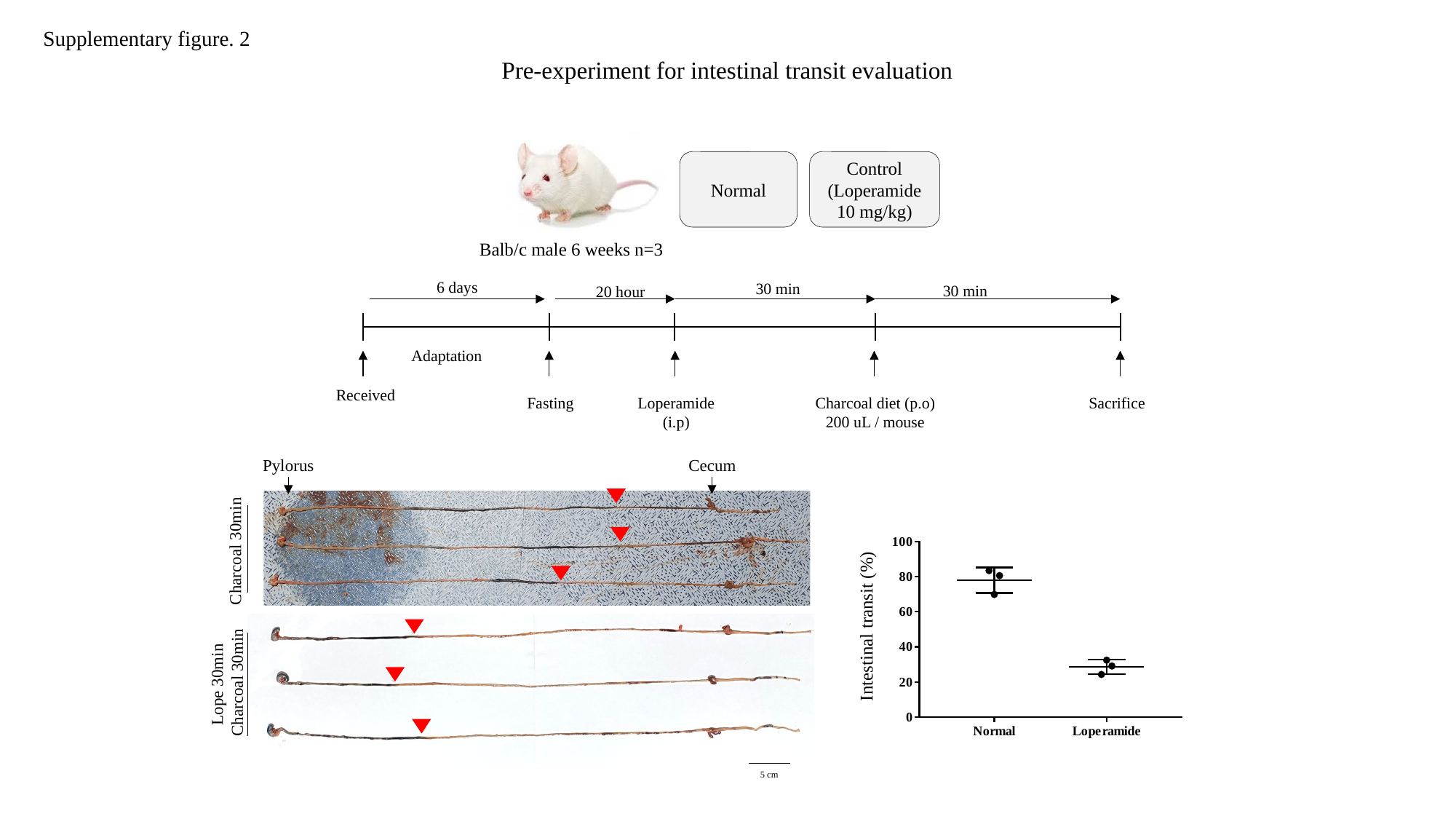

Supplementary figure. 2
Pre-experiment for intestinal transit evaluation
Normal
Control
(Loperamide 10 mg/kg)
Balb/c male 6 weeks n=3
6 days
30 min
30 min
20 hour
Adaptation
Received
Fasting
Loperamide
(i.p)
Charcoal diet (p.o)
200 uL / mouse
Sacrifice
Pylorus
Cecum
Charcoal 30min
Intestinal transit (%)
Lope 30min
Charcoal 30min
5 cm
